# Advances in the Arms Race Between Silkworm and Baculovirus

**DOI:** 10.3389/fimmu.2021.628151

**Published:** 2021-02-09

**Authors:** Liang Jiang, Marian R. Goldsmith, Qingyou Xia

**Affiliations:** ^1^ State Key Laboratory of Silkworm Genome Biology, Southwest University, Chongqing, China; ^2^ Biological Science Research Center, Southwest University, Chongqing, China; ^3^ Department of Biological Sciences, University of Rhode Island, Kingston, RI, United States

**Keywords:** antiviral immunity, baculovirus, *Bombyx mori nucleopolyhedrovirus*, immune evasion, silkworm

## Abstract

Insects are the largest group of animals. Nearly all organisms, including insects, have viral pathogens. An important domesticated economic insect is the silkworm moth *Bombyx mori*. *B. mori nucleopolyhedrovirus* (BmNPV) is a typical baculovirus and a primary silkworm pathogen. It causes major economic losses in sericulture. Baculoviruses are used in biological pest control and as a bioreactor. Silkworm and baculovirus comprise a well-established model of insect–virus interactions. Several recent studies have focused on this model and provided novel insights into viral infections and host defense. Here, we focus on baculovirus invasion, silkworm immune response, baculovirus evasion of host immunity, and enhancement of antiviral efficacy. We also discuss major issues remaining and future directions of research on silkworm antiviral immunity. Elucidation of the interaction between silkworm and baculovirus furnishes a theoretical basis for targeted pest control, enhanced pathogen resistance in economically important insects, and bioreactor improvement.

## Introduction

Insects are globally distributed and play vital roles in the biosphere. Lepidoptera is a major insect taxon with an estimated 150,000 to 180,000 described species (1, 2). Many lepidopterans are pests that adversely affect agricultural production. However, the silkworm moth *Bombyx mori*, the only fully domesticated insect, is an economically important lepidopteran used for silk production in many developing countries (3, 4). China is the largest producer of silkworm cocoons, with an annual value for the output of the silk industry of about 200 billion Yuan (about 30 billion USD) (3). Pathogenic viruses are severe threats to all organisms and silkworm viruses cause losses of almost 16% of potential cocoon production each year. *Bombyx mori nucleopolyhedrovirus* (BmNPV) is a primary silkworm pathogen. This typical baculovirus causes major economic losses in sericulture (3). Baculovirus is also used as a biological control agent against insect pests and as a bioreactor. The Silkworm Genome Project was completed >10 years ago (5–8) and promoted *B. mori* to model insect status in basic and applied research (9). Here, we present a broad overview of silkworm–baculovirus interactions. We also discuss the major challenges and future directions of research in silkworm antiviral immunity.

## Baculovirus Host Invasion Mechanism

Baculovirus consists of a circular double-stranded DNA genome that combines with capsid proteins to form an enveloped nucleocapsid (3, 10). *Autographa californica multiple nucleopolyhedrovirus* (AcMNPV) is a close relative of BmNPV and the most well studied baculovirus (11, 12). Both NPVs are models for basic molecular research which have been used to elucidate the baculovirus infection cycle. The baculovirus replication cycle includes two virion phenotypes, an occlusion-derived virus (ODV) and a budded virus (BV). ODVs are packaged in occlusion bodies and induce host infection, whereas BVs spread throughout the host after infection (12, 13). ODVs and BVs have the same nucleocapsids but different envelopes. BVs mature early during infection and acquire their envelopes from modified host cell membranes. In contrast, ODVs mature late in infection and form their envelopes within host nuclei (14, 15). BVs and ODVs interact differently with host cells: ODVs fuse with the midgut epithelial cell membrane, whereas BVs are internalized by adsorptive endocytosis (15).

**Figure 1:**
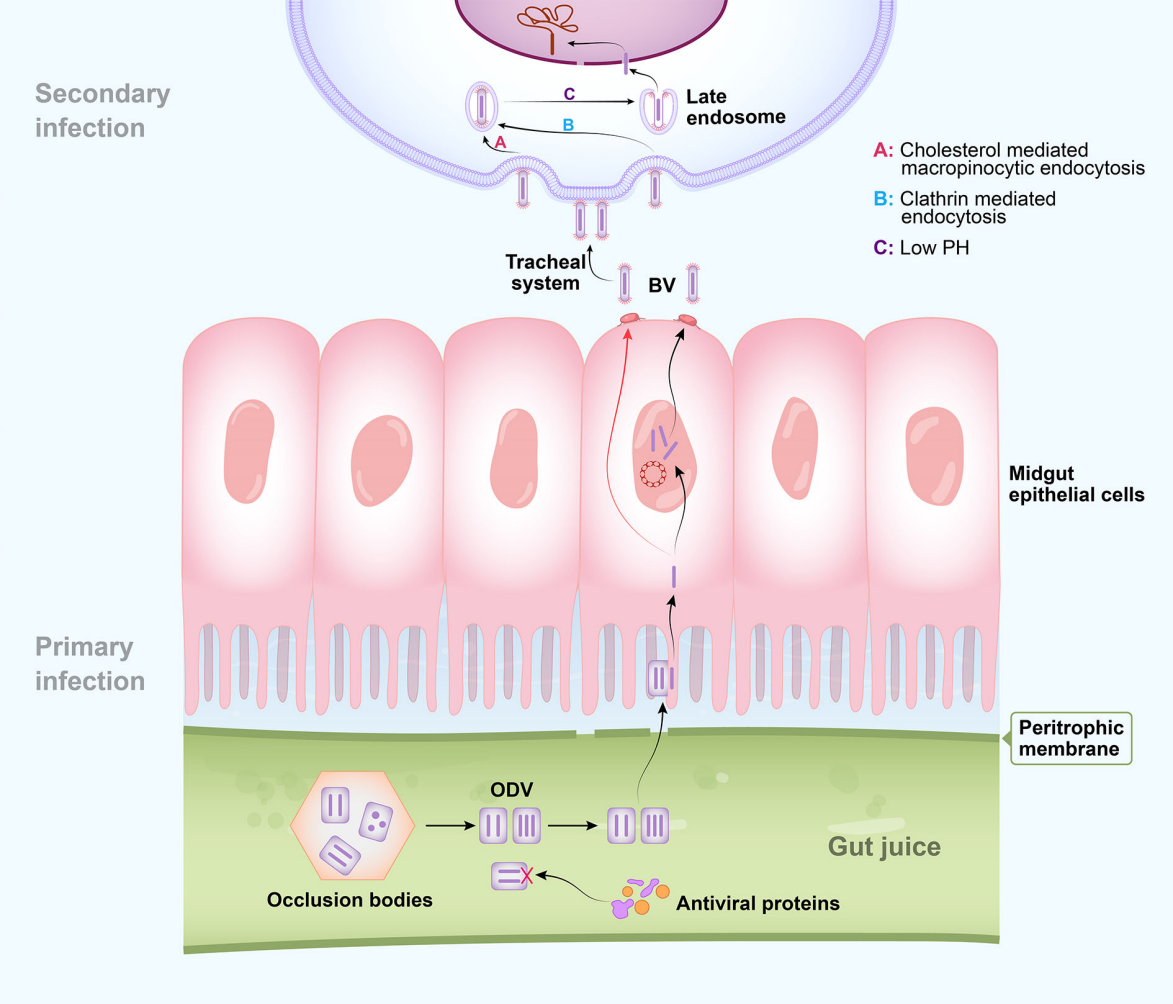
Schematic diagram of baculovirus entry. Occlusion-derived viruses (ODVs) are released from occlusion bodies in the alkaline environment of larval gut juice after ingestion. Several insect gut juice proteins have strong antiviral capacity against ODVs. Intact ODVs pass through the peritrophic membrane and nucleocapsids enter the midgut epithelial cells *via* envelope-mediated membrane fusion to cause primary infection. Progeny budded viruses (BVs) spread through the host *via* the tracheal system to cause secondary infection. Binding and penetration into host cells by BV of both *Bombyx mori nucleopolyhedrovirus* (BmNPV) and *Autographa californica multiple nucleopolyhedrovirus* (AcMNPV) are mediated by the GP64 envelope glycoprotein which is specific to BV. BmNPV BV penetrates nonmidgut host cells by multiple strategies, including clathrin-independent macropinocytic endocytosis mediated by cholesterol on the cell membrane and clathrin- and dynamin-dependent endocytosis pathways. Successful BV entry also requires low pH. Nucleocapsid uncoating in the nucleus results in the subsequent virus infection process.

Baculovirus occurs in the environment in the form of occlusion bodies. For infection, it invades insect larvae mainly by ingestion (3). ODVs are released after these occlusion bodies dissociate in the alkaline environment of larval gut juice. They pass through the peritrophic membrane, invade the midgut, and cause primary infection (**Figure 1**) (11–13). Several envelope proteins known as *per os* infectivity factors (PIFs) are unique to ODVs. They mediate specific ODV binding to midgut columnar epithelial cells and initiate oral infection by binding to receptors (16–19), after which nucleocapsids enter the epithelial cells *via* envelope-mediated membrane fusion (3, 11). Viral DNA is then released from the nucleocapsids and used as a template to generate new DNA and mRNA (3, 11).

Baculoviral gene expression occurs in four phases: immediate early, delayed early, late, and very late. In an infected cell, viral DNA replication starts at 8 h post infection (hpi) and represents the transition from the early stage to the late stage (20, 21). During early infection, host RNA polymerase transcribes the viral DNA and produces the elements required for its replication (15). Viral DNA replication and transcription then form nucleocapsid progeny that acquire envelopes by budding from the host cell membrane. The latter is modified mainly by virally encoded fusion protein GP64 to generate a BV, which causes systemic infection *via* the host tracheal system (13, 22, 23). At the latter infection stages, progeny ODVs acquire envelopes in the nucleus, possibly derived from nuclear membranes modified by several viral proteins (24), are subsequently assembled into occlusion bodies and released into the environment after host disintegration (11, 22).

BmNPV BV utilizes multiple strategies to invade host cells (**Figure 1**). Binding and penetration into host cells by BV of both BmNPV and AcMNPV are mediated by the GP64 envelope glycoprotein which is specific to BV (12, 25, 26). GP64 contains a cholesterol recognition amino acid consensus (CRAC) domain which is known to be essential for fusion between the BV envelope and mammalian cell membrane (26, 27). The BmN and BmE cell lines are derived from the ovary and embryonic cells of silkworm, respectively. Various endocytic inhibitor assays disclosed that BmNPV BV penetrates BmN cells by clathrin-independent macropinocytic endocytosis mediated by cholesterol on the cell membrane (28). The cholesterol transporter BmNPC1 interacts with GP64. Its deficiency inhibits viral penetration rather than viral binding to BmE cells (29). In contrast, BmNPV BV uses clathrin- and dynamin-dependent endocytosis pathways to penetrate BmN cells. Successful BV entry also requires low pH (25). A number of studies were performed to identify the host receptor of GP64 (12). The membrane protein BmREEPa is not a direct NPV receptor but interacts with GP64 and may participate in BV attachment or binding (30). Yeast two-hybrid and coimmunoprecipitation (Co-IP) assays demonstrated that the silkworm protein SINAL10 binds GP64, is concentrated near the cell membrane, and stimulates BmNPV proliferation in BmN cells (14). Nevertheless, to date, unequivocal identification of a receptor for GP64 remains elusive (12).

Baculovirus encodes some auxiliary genes to enhance its infection in insect larvae, including viral fibroblast growth factor (*vfgf*), ecdysteroid (UDP)-glucosyltransferase (*egt*), and *p35* (31). Horizontal gene transfer (HGT) between host and pathogen might augment pathogen survival and propagation. Several BmNPV auxiliary genes were acquired from the silkworm genome *via* HGT. These include *egt*, *vfgf*, and protein tyrosine phosphatase (*ptp*) (32). BmNPV PTP is a virus-associated structural protein which might have originated from insect *ptp-h* (32). Deleting it reduces production of progeny in larval silkworm hosts; moreover, the mutation can be rescued by inserting *Bmptp-h* into BmNPV ptp-deleted virus (33), and overexpression of *Bmptp-h* accelerates BmNPV multiplication in BmE host cells (34). Other experiments involving deletion and insertion of *ptp* and *egt* (34, 35) showed that HGT-derived genes are dispensable for virus production in certain cell lines but affect progeny contents and may control host physiology.

## Silkworm Immune Response to Baculovirus

**Figure 2:**
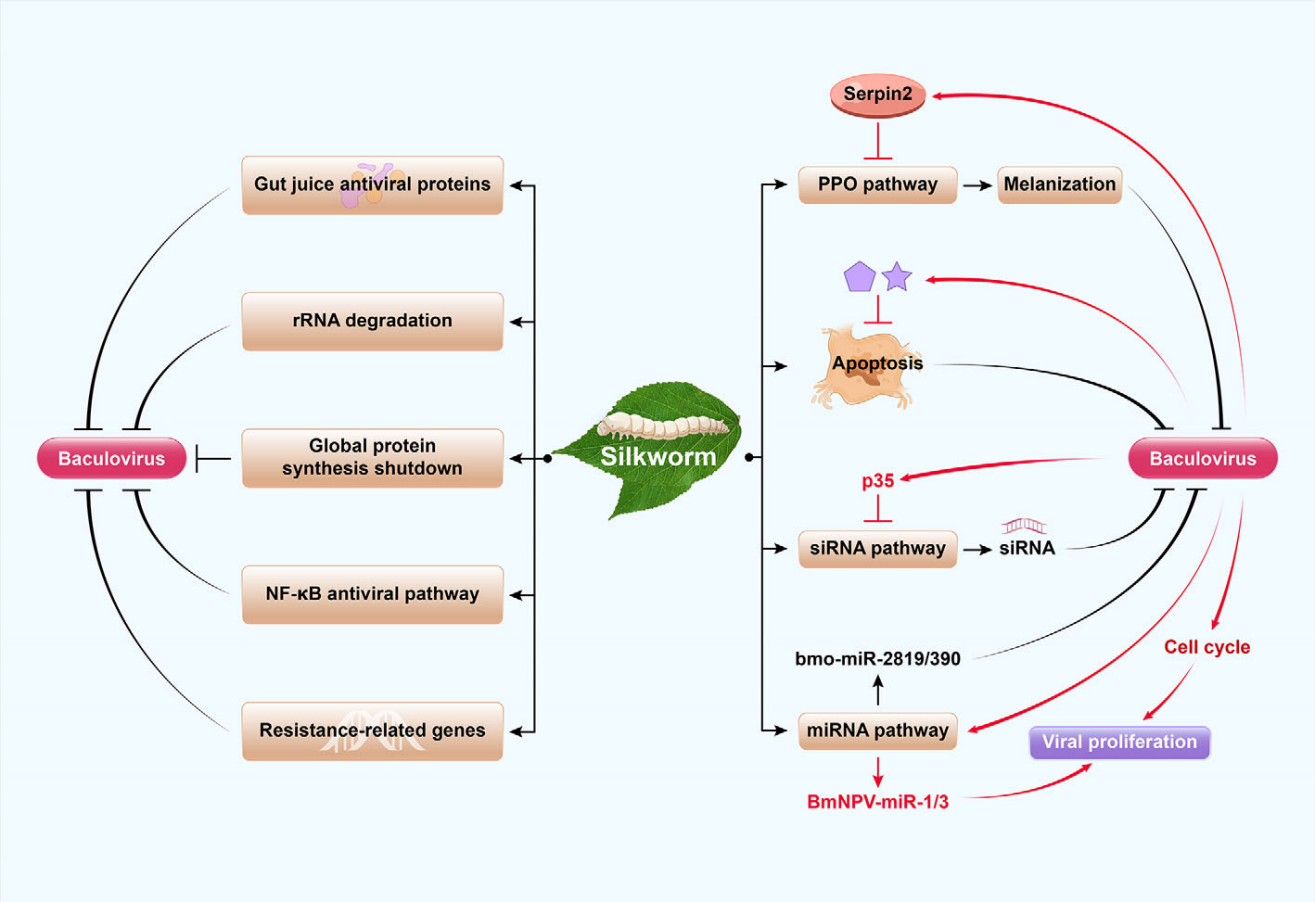
Model of the arms race of silkworm and baculovirus. Silkworms have several antiviral immune responses which they use against baculovirus infections. These include global protein synthesis shutdown, rRNA degradation, inactivation by gut juice antiviral proteins, host gene-encoded resistance, NF-κB antiviral pathway, apoptosis, melanization, and RNAi-based antiviral response. The prophenoloxidase (PPO) activation cascade causes melanization to block baculovirus infection, which is negatively regulated by serpins. RNAi antiviral defense of insects includes the major mechanism of the siRNA pathway and the minor contribution of the miRNA pathway. The silkworm-encoded miRNA bmo-miR-2819 and bmo-miRNA-390 inhibit BmNPV proliferation by downregulating viral genes. As a confrontation, baculovirus have developed several strategies to escape host immunity and promote their own replication and proliferation, including inhibition of antiviral apoptosis, melanization, RNAi and regulation of the cell cycle. For example, *Bombyx mori nucleopolyhedrovirus* (BmNPV) induces *Bmserpin2* to inhibit host melanization. Meanwhile, *Autographa californica multiple nucleopolyhedrovirus* (AcMNPV) p35 inhibits siRNA pathway. Additionally, baculoviruses exploit the miRNA pathway to encode their own miRNAs (such as BmNPV-miR-1 and BmNPV-miR-3) for viral propagation.

Innate immune responses in insects control and clear pathogens following infection (36, 37). Lepidopteran insects have several antiviral immune responses which they use against baculovirus infections. These include global protein synthesis shutdown, rRNA degradation, inactivation by gut juice antiviral proteins, melanization, apoptosis, RNAi-based antiviral response, and host gene-encoded resistance (**Figure 2**) (3, 36–39). Among these immune responses, there are relatively few studies on the mechanisms of the first two processes. After AcMNPV infection of *B. mori* cells, rRNA degradation is triggered by six amino acid residues (positions 514 and 599) of viral protein P143 as a primary antiviral response. Global protein synthesis shutdown then follows viral DNA replication, resulting in abortive infection (38, 40). The latter processes are more clearly delineated, and each process is described in turn here.

The insect midgut is the first tissue to be infected after baculovirus ingestion. Hence, it is an important immune organ which acts as a first line of defense against pathogens (41, 42). Several insect gut juice proteins secreted from the midgut have strong antiviral capacity. The antiviral proteins Bmlipase-1 (43), BmSP-2 (44), BmNOX (45), red fluorescent proteins (RFPs) (46), Bmtryp (47), and BmLHA (48) have been isolated from silkworm larva gut juice, which inhibit BmNPV at an initial infection stage. The activation of energy synthesis by adenosine signaling following baculovirus infection is a physiological response in the silkworm that supports its innate immunity (49). Melanization is a prominent humoral response in insects. It consists of a cascade of clip-domain serine proteases (cSPs) that converts zymogen prophenoloxidase (PPO) into active phenoloxidase (PO), which is negatively regulated by serpins. PO catalyzes melanin formation to encapsulate and kill invading pathogens (50, 51). Baculovirus infection is efficiently blocked by the PPO activation cascade (50). *Bmserpin2* knockdown increases PO activity and decreases viral DNA content in silkworm haemolymph infection with BmNPV (52). The stage of infection at which melanization inhibits baculovirus infection needs further exploration.

**Figure 3:**
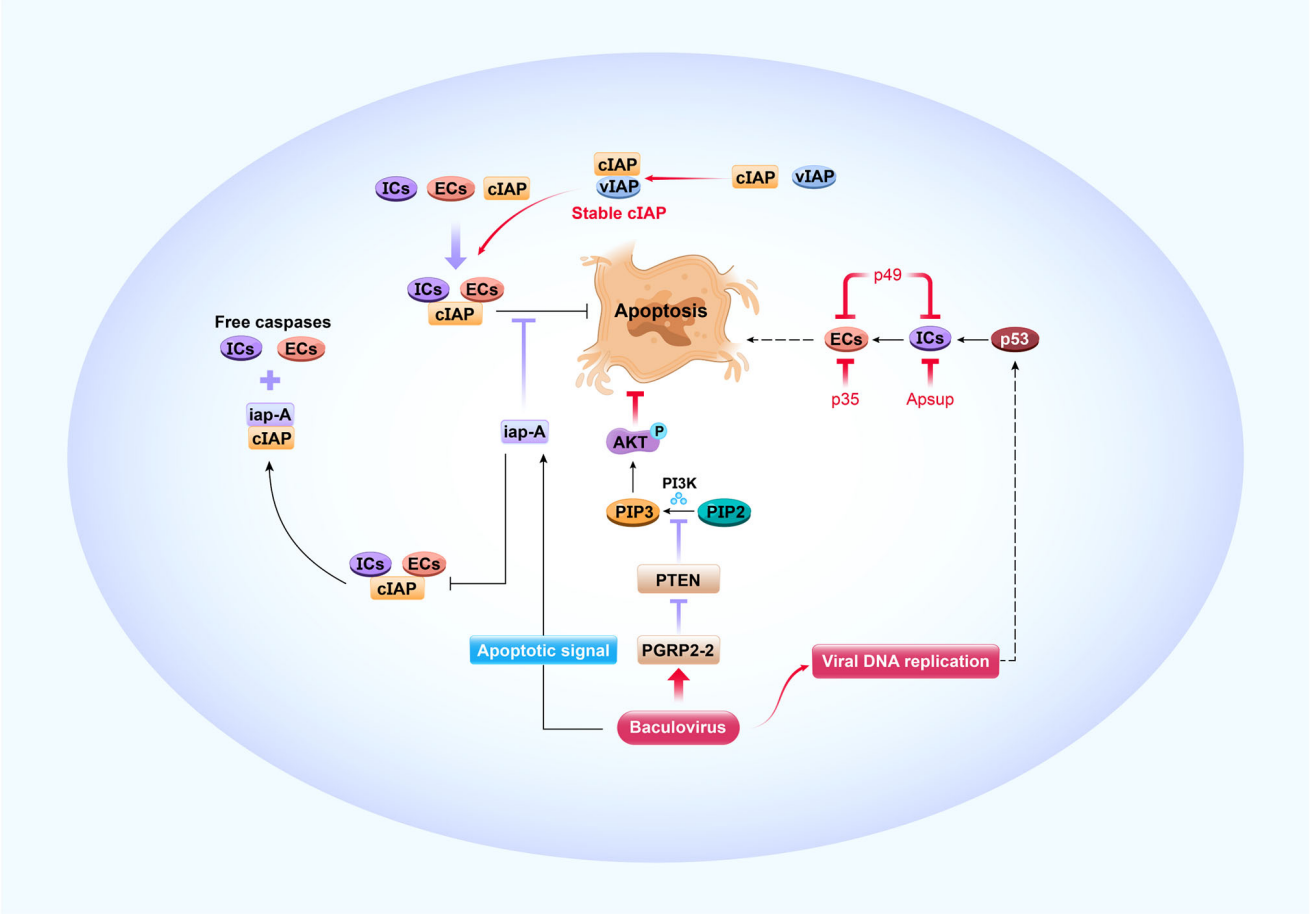
Antiviral apoptosis and its modification by baculoviruses. The apoptotic caspase cascade comprises upstream initiator caspases (ICs) and downstream effector caspases (ECs). The cellular inhibitor of apoptosis (cIAP) binds caspases and blocks apoptosis in normal cells. Apoptotic signaling is initiated upon baculovirus infection, which causes iap-Antagonist (iap-A) to bind cIAP and release free caspases that facilitate apoptosis. Viral DNA replication triggers host p53 pro-apoptosis, which accelerates IC and EC activity. Progression of antiviral apoptotic signaling cascades is prevented by baculovirus-encoded apoptosis suppressors such as viral IAP (vIAP), p35, p49, and Apsup. When the apoptotic signal is initiated, vIAP blocks apoptosis by interacting with unstable cIAP such that the cIAP levels and antiapoptotic activity are maintained. Viral p35 binds ECs and p49 binds ICs and ECs to block apoptosis. Apsup inhibits apoptosis by preventing IC activity. BmNPV induces the pattern recognition receptor protein *PGRP2-2* to suppress *PTEN* and prevent it from inhibiting PI3K/Akt signaling and activating p-Akt. In this manner, cell apoptosis is inhibited. The resultant increase in cell survival is conducive to NPV proliferation.

Apoptosis is a genetically controlled process that removes unwanted or damaged cells. It serves as an important antiviral defense mechanism in insects (15, 37, 53–55). The apoptotic caspase cascade comprises upstream initiator caspases (ICs) and downstream effector caspases (ECs) (15, 53) (**Figure 3**). To initiate apoptosis, ECs are activated by ICs, and then cleave other signaling proteins (56). In lepidopterans, caspase-1, caspase-2, and caspase-3 are ECs while caspase-5 (Dronc) and caspase-6 (Dredd) are ICs (57). A cellular inhibitor of apoptosis (IAP) binds caspases, blocks their function, and prevents apoptosis activation in normal cells (15, 58). In BmN cells, *B. mori* iap1 (BmIAP1) interacts with BmDronc and Bmcaspase-1 and downregulates apoptosis (58). Apoptotic signaling, which is initiated upon baculovirus infection, promotes iap-antagonist (iap-A) binding to cellular IAP and releases free caspases to facilitate apoptosis (15, 53). The host p53 protein is pro-apoptotic and triggers antiviral apoptosis upon viral DNA replication. It elevates caspase-3-like protease activity and enhances BmDronc processing in BmN cells after BmNPV infection (53) (**Figure 3**). Nevertheless, a DNA damage response, which is elicited upon viral DNA replication, depletes cellular IAP protein, activates apoptosis, and promotes baculovirus multiplication in infected cells (59–61). Although apoptotic pathways and their associated viral and cellular factors play important roles in regulating the outcome of baculovirus infection in insect cells, their mechanisms and interactions are complex and remain to be fully elucidated.

RNA interference (RNAi) is an ancient post-transcriptional antiviral regulatory process in insects (36, 62) whereby the host RNAi response degrades baculovirus transcripts (63). In this process, viral infections generate dsRNAs that trigger the RNAi machinery and process them into viral short interfering RNAs (vsiRNAs) that target viral RNA sequences and inhibit viral proliferation (64). Another RNAi response involves the microRNA (miRNA) pathway in which precursor miRNA (pre-miRNA) is cleaved into mature miRNA that regulates gene expression by targeting specific mRNAs (65). Cellular miRNAs also affect viral infections and play important roles in host–pathogen interactions. The silkworm-encoded miRNA bmo-miR-2819 is upregulated at the delayed early stage in infection, and its overexpression inhibits BmNPV proliferation by downregulating viral *ie-1* (66). Similarly, bmo-miRNA-390 downregulates the expression of BmNPV-*cg30* (67). The PIWI-associated RNA (piRNA) pathway is also involved in an antiviral response but little information is reported in silkworm (68). Results from published reports reveal that the siRNA pathway is the major mechanism, whereas the contribution of the miRNA pathway is minor in RNAi antiviral defense of insects (**Figure 2**).

Innate immune signaling pathways and resistance-related genes play an important role in antiviral defense. The Imd and Toll signaling pathways participate in the antiviral immune response (36, 54) but do not seem to play roles in the silkworm BmNPV response. BmNPV infection induces cGAMP production in BmE cells and BmSTING responds to the cGAMP and activated Dredd caspase-mediated NF-κB antiviral signaling pathways (69). Antimicrobial peptides (AMPs), humoral immunity, and reactive oxygen species (ROS) may also be involved in the antiviral response (45, 70). Dozens of candidate genes regulating the silkworm immune response to baculovirus have been screened *via* multi-omics using various resistant hosts. However, the functions of only a few of them are verified in cells or individuals. For example, the BmLHA level in the digestive juice of resistant silkworm strains is relatively higher than that of susceptible silkworms, and recombinant BmLHA inhibits BmNPV proliferation in silkworm larvae (48). Similarly, *BmAtlastin-n* is highly expressed in resistant BmE-SWU2 cells but not in BmE-SWU1 cells susceptible to BmNPV, and *BmAtlastin-n* overexpression inhibits BmNPV reproduction in BmE-SWU1 cells and transgenic silkworms (71). Additionally, *B. mori* heat shock protein 19.9 (*Bmhsp19.9*) is upregulated at the late stage after BmNPV challenge in BmE cells and silkworms, and its overexpression markedly inhibits BmNPV proliferation in the hosts (72). Finally, overexpression of lysozyme *BmC-LZM*, which is upregulated at the very late stage of BmNPV infection in BmE cells, inhibits BmNPV virus in BmE cells but does not decrease mortality in silkworm larvae (73). The anti-BmNPV mechanisms of the aforementioned resistance-related genes are unclear and merit further investigation.

## Viral Immune Evasion Mechanism

Viruses have developed several strategies to escape host immunity and promote their own replication and proliferation, including inhibition of antiviral melanization, autophagy, apoptosis, RNAi and regulation of the cell cycle (**Figure 2**). Baculoviruses can suppress host melanization so that they can proliferate. Several SPs (serine proteases) and their homologs are upregulated in response to bacterial or fungal challenge but downregulated in response to baculovirus infection (50, 51). For example, when serpins 5 and 9 are induced by HearNPV in *Helicoverpa armigera*, they inhibit SPs and melanization and promote viral infection (51). Similarly, *Bmserpin2* is upregulated and PO activity is diminished in haemolymph following BmNPV infection in silkworm. Hence, BmNPV inhibits host melanization by regulating *Bmserpin2* expression (**Figure 2**) (52). Additionally, several potential resistance-related genes such as *BmPP2A* (74) and *BmPEPCK-2* (75) are downregulated by BmNPV to allow robust viral proliferation.

Autophagy is a catabolic biological process in the body, which has antiviral efficacy by targeting viruses and sending them to the lysosome for phagocytosis and degradation. At the same time, viruses can also use autophagy to enhance their own replication (76). However, little is known about the association between BmNPV and autophagy. *Atg 6*, *Atg 7*, *Atg 8*, and *Atg 13*, proteins involved in various stages of autophagy, are all upregulated in BmN-SWU1 cells (77) but downregulated in BmE cells (75) following BmNPV infection, possibly because of the relative differences among cell lines and internal reference genes used in these experiments. Understanding the roles and mechanisms of such immuno-suppressive processes during BmNPV infection is clearly important for future applications to enhance their impact (for pests) or protecting their hosts (for beneficials) and merit further examination.

Baculoviruses can inhibit host antiviral apoptosis through a variety of strategies (**Figure 3**). The progression of apoptotic signaling cascades is prevented by virus-encoded apoptosis suppressors such as viral IAPs, p35, p49, and Apsup (55, 78, 79). Six IAPs (*iap1-6*) have been identified in baculoviruses that inhibit apoptosis in insects (78–80). Unlike their cellular counterparts, they lack an N-terminal instability motif (81) and stabilize cellular IAPs (82). In a model mechanism, Op-IAP3 derived from OpMNPV blocks apoptosis by interacting with an unstable auto-ubiquitinating host IAP such that cellular IAP levels and antiapoptotic activity are maintained (82). Similarly, IAP1 and IAP2 from BmNPV interact with BmIAP, and both BmIAP and viral IAPs increase BmNPV proliferation in infected silkworm cells (80). Numerous studies have shown that viral protein p35 blocks apoptosis by binding ECs (79, 83, 84), and p49 protein binds ICs and ECs and blocks apoptosis (85, 86). Additionally, Apsup from LdMNPV inhibits apoptosis by preventing proteolytic Dronc (IC) processing (87). Recently, our research demonstrated that peptidoglycan recognition protein (PGRP) is regulated by virus to inhibit host antiviral apoptosis, which is well known to recognize invading bacteria and fungi to activate host immune defenses (54). For example, BmNPV induces *BmPGRP2-2* to suppress *PTEN* and the inhibition of PI3K/Akt signaling, increase p-Akt production and activation, and inhibit cell apoptosis (54). Clearly, enhanced host cell survival is beneficial for viral proliferation (**Figure 3**).

Viruses have evolved strategies to circumvent host antiviral RNAi (siRNA and miRNA pathways). Almost all plant viruses and some insect viruses encode viral suppressors of RNAi (VSRs) to counteract the host siRNA pathway and inhibit vsiRNA production (88, 89). AcMNPV p35 is responsible for the suppression of RNAi in various insect cells; its VSR activity acts downstream in the RNAi pathway and is not associated with its antiapoptotic activity (89). The identification of BmNPV VSRs and clarification of their modes of action require further research. On the other hand, it is evident that baculoviruses exploit the miRNA pathway for their own propagation, suppress cellular miRNAs after infection, encode their own miRNAs, and disrupt host defense mechanisms that interfere with viral propagation (90–92). For example, BmNPV-miR-1 suppresses host miRNA biogenesis by regulating the exportin-5 cofactor Ran and enhancing viral multiplication (92). Simultaneously, BmNPV-miR-3 facilitates viral infection by modulating the expression of P6.9 and other late BmNPV genes (91) (**Figure 2**). Several miRNAs have been predicted in the BmNPV genome; however, only four miRNAs (BmNPV-miR-1, BmNPV-miR-2, BmNPV-miR-3, and BmNPV-miR-4) have been empirically identified (90) and biological functions of only two miRNAs have been uncovered thus far. Deciphering viral miRNA targets and functions remains a challenging task.

Virus regulation of the host cell cycle might be an important immune evasion strategy and could promote its proliferation. The normal insect cell life cycle is characterized by a complex series of events ranging from cell growth to replication, but this process is disrupted during infection (15). Baculovirus infection arrests the cell cycle at S or G2/M. The AcMNPV protein EC27 arrests the host cell cycle in the G2/M phase, and this arrest enables ODV maturation (93, 94). ERK regulates cell proliferation, differentiation, and apoptosis and is conserved among different species (95). The ERK signaling pathway is activated during the late phase of BmNPV infection *via* the *B. mori* epidermal growth factor receptor (BmEGFR). The latter inhibits cell proliferation and increases viral replication by increasing the G2/M phases of the cell cycle (96). *BmSpry* is a negative feedback regulator of the BmEGFR-ERK cascade; its inhibitory activity is upstream of ERK. It is downregulated by BmNPV to elevate ERK phosphorylation (p-ERK), thereby enhancing viral reproduction (95, 97). The modification mechanisms of cell cycle phases during baculovirus infection are only partially elucidated and need more experimentation.

## Enhancement of Host Antiviral Capacity

No fundamental strategies have been established to cope with BmNPV during sericulture; instead, this industry mainly relies on thorough disinfection and strict breeding operation techniques to prevent virus infectivity. Breeding resistant host insect strains would help contend with baculovirus infection in sericulture (3, 98, 99). However, enhancing pathogen resistance in the host is usually accomplished at the expense of economically important traits, which is a major constraint in traditional silkworm breeding methods. This compromise may be avoided by applying transgenic and gene editing techniques (3). The antiviral capacity of transgenic silkworms could be enhanced using strategies based on the BmNPV infection process such as inhibiting BmNPV at the initial infection stage *via Bmlipase-1* overexpression (100), targeting BmNPV mRNA with RNAi (21), inhibiting BmNPV protein synthesis by *hycu-ep32* overexpression (101), and suppressing BmNPV by regulating the host immune pathway (54). Antiviral capacity could be further increased by optimizing and integrating the aforementioned anti-BmNPV strategies (41, 42, 102). Transgenic CRISPR/Cas9 system-mediated mutagenesis randomly targeting and inactivating the viral genome has been studied as a potential approach against BmNPV infection in silkworm (103). Theoretically the inhibitory effect of the CRISPR/Cas9 system (knock out) on the virus should be higher than that of the RNAi system (knock down) when targeting the same viral genes. However, silkworms with inserted DNA fragments expressing dsRNA (21) or gRNA (103) are all transgenic strains and security assessment is an unavoidable challenge under the conditions of mass rearing practiced in sericulture.

Several drugs have been evaluated for their antiviral activity against BmNPV. The bacterial secondary metabolite prodigiosin inhibits BmNPV in BmN cells and is a potential antiviral compound (104). However, its antiviral efficacy must be tested in insect larvae. The single-crystal compound seselin extracted from *Aegle marmelos* (a kind of citrus fruit) shows antiviral activity against BmNPV in silkworm larvae (105). AZD8835, AMG319, HS173, AS605240, GDC0941, BEZ235 are PI3K inhibitors and afuresertib is an Akt inhibitor. These seven drugs target the PI3K/Akt pathway to decrease p-Akt and all inhibit BmNPV in BmE cells; nevertheless, only AMG319 and AZD8835 inhibit viral proliferation in silkworm larvae. Of these two, AZD8835 exhibits a stronger antiviral efficacy which might be due to lower drug toxicity in larvae and stronger inhibition of p-Akt (106). The development of drugs with high antiviral capacity in silkworms could decrease mortality in sericulture. However, their absorption and utilization efficiency, inhibitory efficacy, and cost-effectiveness must be increased while their cytotoxicity is decreased (106).

## Major Issues in Silkworm Antiviral Studies

Several conflicting results have been reported for the same genes in previous studies on the interaction between silkworm and baculovirus. These discrepancies may be explained by the use of different silkworm strains and cell lines as well as inappropriate internal reference genes (RGs). RGs must not be affected by experimental conditions and should be expressed at the same constant level in all samples. Unsuitable RGs lead to the incorrect interpretation of gene expression patterns and functions (107). As a widely used example, actin participates in baculovirus proliferation and expression after viral infection in silkworms (107). Hence, *actin* cannot serve as the RG for mRNA and protein detection in studies involving the interaction between silkworms and viruses. In contrast, *TIF-4A* is an appropriate RG for gene expression analysis (107) and GAPDH (54) is an appropriate internal reference for protein content measurements following viral challenges in silkworms.

Transgenic silkworms with high antiviral capacity have been constructed (102, 103, 108, 109). Nevertheless, their commercial application still faces great challenges. Security assessment must be performed on transgenic silkworms before they are commercialized (3). There are operational guidelines for safety assessments of genetically modified (GM) vertebrates and plants but not for insects, including silkworms. Thus, safety evaluations are difficult to execute on transgenic silkworms. Based on GM animal safety assessment guidelines, we conducted a preliminary evaluation of transgenic silkworms in our laboratory. A classical genetic analysis and molecular characterization of 11 successive generations showed that an inserted foreign DNA fragment was stably inherited in transgenic silkworms (110). The disposition of the inserted DNA in transgenic silkworms fed to chickens was also examined, with no apparent transfer of transgenic DNA from silkworms to chickens (111). A subacute toxicity test comprising a 28 d feeding study in rats showed that transgenic silkworms are toxicologically equivalent to normal silkworms and are safe for rats (112). Transgenic silkworms are unable to survive and reproduce in the field and would not cause environmental risks of competition with other insects, and no interspecific hybridization of transgenic silkworms and *Bombyx mandarina* was observed in nature, so transgenic silkworms have no risks to biodiversity (113). The transgenic silkworms that produce green fluorescent silk have been reared in a sericulture farm in Japan since 2017 (113). Nevertheless, the design of safety assessment procedures and identification of transgenic antiviral silkworm indicators are urgently required as they cannot be the same as those already implemented for GM vertebrates. A notable difference in appropriate safety assessment design is that although GM vertebrates are used for food and feed, transgenic silkworms are used only in silk production.

## Future Directions of Silkworm Antivirus Research

Current research on the mechanisms by which baculovirus penetrates its host has focused mainly on BVs and insect cell lines (25, 28–30). Some of the constraints of investigations into the interactions between individual insects and baculovirus include limitations in insect genetic manipulation, long experimental periods, and intensive labor. The PIFs of ODV envelopes form complexes that mediate viral invasion in the insect midgut (16–19). The receptors involved in ODV invasion may also be part of a complex. Screening and identifying ODV receptor genes in the silkworm midgut are difficult exercises. The process of ODV entry must first be clarified in order to develop methods to block BmNPV infection in silkworm. Earlier studies reported that the resistance of silkworms to BmNPV is controlled by major genes and modified by minor genes (98); however, a major resistance gene has not yet been identified despite numerous attempts using various methods. Identification of resistance genes and analysis of silkworm antiviral mechanisms against BmNPV merit further investigation. In future experiments, we will screen for negative regulatory factors in the immune pathway using genome-wide CRISPR (114) and identify the host proteins that bind the virus by use of inhibitors. The target genes will be knocked out *via* gene editing to improve silkworm resistance. Immune priming is a new strategy to increase host antiviral capacity (115, 116) and we will clarify its mechanism of action in silkworm. The influences of gut microbes, heat shock response, and DNA methylation on viral silkworm infections will also be evaluated.

The baculovirus expression vector system (BEVS) is a bioreactor for the production of recombinant proteins and vaccines. Several vaccines produced by BEVS have been approved for human and/or veterinary use (15, 117, 118). The BEVS was invented using AcMNPV in combination with an insect cell system (117). However, the cost of silkworm rearing is much lower than that of insect cell culture, promoting the use of BmNPV to generate foreign proteins using silkworm larvae as bioreactors. Understanding the baculovirus infection mechanism including modification of host and viral proteins will facilitate application of a combined BmNPV-silkworm system in production of high value-added medical proteins. Explorations of the silkworm immune response to baculovirus will help construct silkworms less sensitive to BmNPV by inhibiting the host immune system and resistance genes, and in combination with BmNPV with attenuated virulence, further reduce the costs of foreign protein fabrication.

Baculoviruses have been applied worldwide as biopesticides for the control of various insect pests (119, 120). Compared to chemical pesticides, baculoviruses are environmentally safe. Nevertheless, their killing rates are low, and their host range is narrow (15, 31, 119). In the future, baculovirus should be modified to expand its target pest host range. Its antagonism against the host immune defense must be strengthened by accentuating viral host immune evasion mechanisms which will enable use of lower viral titers to kill pests faster. Less sensitive insect bioreactors for baculovirus-based biopesticides should be designed to reduce production costs. Further investigations into silkworm antiviral mechanisms will provide a reverse theoretical basis and reference for biological insect pest control.

## Conclusion

Viruses exert strong selection pressure on their hosts to evolve resistance pathways. In turn, these genetic modifications enable viruses to escape host antiviral mechanisms. This arms race favors host defense diversification and the development of viral escape mechanisms (37). Several factors contribute to viral coevolution with its natural host. A complete elucidation of antiviral immunity and immune evasion is challenging as numerous complex pathways are involved (37). Hence, BmNPV research should focus on actual silkworms rather than cell lines and novel technologies such as gene editing and value-added protein biosynthesis. Studies involving the silkworm–baculovirus model are highly informative as they disclose original antiviral strategies, immune evasion mechanisms, and weaknesses of viruses. In this way, genetic antiviral improvement of silkworms may be achieved along with the development of more effective approaches to control lepidopteran and other insect pests. These applications, along with the realization of more productive and efficient bioreactors for novel baculovirus-insect-derived products, are promising applications for the future.

## Author Contributions

LJ: analyzed data, drew figure, drafted the article, and supervision. MG: review and editing. QX: supervision. All authors contributed to the article and approved the submitted version.

## Conflict of Interest

The authors declare that the research was conducted in the absence of any commercial or financial relationships that could be construed as a potential conflict of interest.

## Abbreviations

AcMNPV, *Autographa californica multiple nucleopolyhedrovirus*; AMP, antimicrobial peptide; *B. mori*, *Bombyx mori*; BEVS, baculovirus expression vector system; BmEGFR, *B. mori* epidermal growth factor receptor; *Bmhsp19.9*, *B. mori* heat shock protein 19.9; BmNPV, *Bombyx mori nucleopolyhedrovirus*; BV, budded virus; Co-IP, coimmunoprecipitation; CRAC, cholesterol recognition amino acid consensus; cSPs, clip-domain serine proteases; ECs, effector caspases; egt, ecdysteroid (UDP)-glucosyltransferase; GM, genetically modified; HGT, horizontal gene transfer; hpi, h post infection; IAP, inhibitor of apoptosis; iap-A, iap-antagonist; ICs, initiator caspases; miRNA, microRNA; ODV, occlusion-derived virus; PGRP, peptidoglycan recognition protein; PIF, *per os* infectivity factor; piRNA, PIWI-associated RNA; PO, phenoloxidase; PPO, prophenoloxidase; pre-miRNA, precursor miRNA; ptp, protein tyrosine phosphatase; RFPs, red fluorescent proteins; RGs, reference genes; RNAi, RNA interference; ROS, reactive oxygen species; siRNAs, short interfering RNAs; SPs, serine proteases; vfgf, viral fibroblast growth factor; VSRs, viral suppressors of RNAi.
